# Vertical Compositional Heterogeneity Induces Instability in All-Inorganic CsPbIBr_2_ Perovskites

**DOI:** 10.1021/acsaem.4c01898

**Published:** 2024-10-08

**Authors:** Paheli Ghosh, Ben F. Spencer, Lethy Krishnan Jagadamma

**Affiliations:** † Energy Harvesting Research Group, School of Physics & Astronomy, SUPA, University of St Andrews, St Andrews KY16 9SS United Kingdom; ‡ Henry Royce Institute and Department of Materials, University of Manchester, Oxford Road, Manchester M13 9PL, United Kingdom

**Keywords:** all-inorganic perovskites, metallic Pb, antisolvent, XPS, HAXPES, vertical heterogeneity, photovoltaics, stability

## Abstract

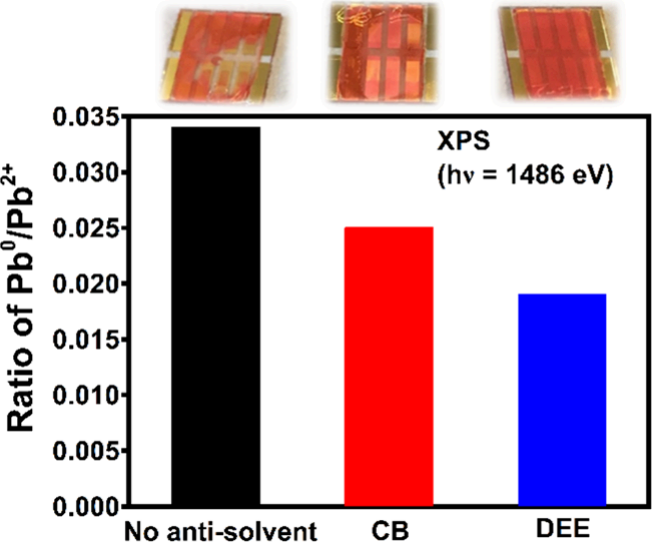

Understanding the vertical compositional homogeneity and defect distribution is of paramount importance in elucidating and maximizing the performance of halide-perovskite-based optoelectronic devices. This work reports the depth-dependent study of the chemical composition and metallic Pb^0^ content of all-inorganic CsPbIBr_2_ perovskite films undertaken using lab-based hard X-ray photoelectron spectroscopy and soft X-ray photoelectron spectroscopy. The presence of elemental or metallic Pb (Pb^0^), in the bulk and at the surface of the perovskite films highlights the formation of defect or recombination centers throughout the analyzed depth. The Pb^0^ content was found to be of higher concentration in the bulk of the CsPbIBr_2_ films compared to that at the surface. Engineering the CsPbIBr_2_ film growth using appropriate antisolvents resulted in the overall reduction and/or complete elimination of Pb^0^ at the surface and at the bulk of the perovskite films. However, the effect of antisolvent treatment was significantly pronounced in the bulk-like region as compared to that at the surface. Pb^0^ is synonymous with defect states/recombination centers in perovskite films and this reduction in defect density due to the antisolvent treatment corroborates the enhanced phase stability and improved solar cell performance of the corresponding CsPbIBr_2_ devices.

## 1 Introduction

Immense research interest in halide perovskite photovoltaic devices has seen a surge in power conversion efficiency from 3.8%^1^ in 2009 to 26.7%^2^ in just over a decade due to their excellent optoelectronic properties and cost-effective fabrication methods.^3−5^ Inorganic perovskite semiconductors such as CsPbX_3_ (where X = Cl, Br, or I) are particularly promising due to their high thermal stability and bandgap tuning via composition engineering (1.73 eV for CsPbI_3_ to 2.3 eV for CsPbBr_3_).^6,7^ Despite this high-temperature tolerance, Cs-based perovskite solar cells (PSCs) with high iodide content show phase instabilities, with the black, cubic, photoactive α-phase destabilizing into the yellow, nonphotoactive, orthorhombic δ-phase at room temperature. Incorporating Br^–^ ions to replace some of the I^–^ ions is an effective strategy for not only tuning the bandgap but also enhancing the stability of the CsPbX_3_ perovskite semiconductors such as in CsPbIBr_2_. The CsPbIBr_2_ perovskite semiconductor with a bandgap of ∼2.05 eV^8^ has found applications in various optoelectronic devices including light emitting diodes,^9^ photodetectors,^10−13^ indoor photovoltaic devices,^14^ integration in tandem devices, and smart photovoltaic windows.^15^ The authors have recently demonstrated the application of high-quality CsPbIBr_2_ films for improved indoor photovoltaic device performance and stability with prospects of powering the next-generation Internet of Things (IoT) sensors.^14^ Formation of highly crystalline CsPbIBr_2_ perovskite crystals along with the reduction in grain misorientation and defect density in the form of Pb^0^ were identified as crucial factors for enhanced photovoltaic performance and stability, however, the distribution of Pb^0^ as a function of depth and understanding of its role in controlling the CsPbIBr_2_ perovskite phase stability remained elusive. The CsPbIBr_2_ perovskite has been particularly selected by authors for this study because of two reasons (i) its suitable bandgap for indoor photovoltaics, where theoretical studies have shown that the optimum bandgap is 1.9–2 eV to maximize the indoor light harvesting^16^ and (ii) enhanced stability of all-inorganic halide perovskites under strong X-rays such as synchrotron-based hard X-ray photoemission spectroscopy (HAXPES) as previously demonstrated.^17^

Previous studies have shown that the presence of Pb^0^ in perovskite films can hinder the crystallization, reduce trap activation energy and form deep-level defect states resulting in the formation of nonradiative decay channels and shunting paths for accelerated degradation.^18−21^ The contribution of Pb^0^ towards the formation of sub-bandgap electronic trap states which are detrimental to perovskite photovoltaic device performance also cannot be ignored.^22^ These studies, along with the recent publication by the authors^14^ show the necessity of developing further understanding of the Pb^0^ defects, especially along the vertical direction of film formation, since charge transport takes place vertically in most of the optoelectronic devices such as photovoltaics, photodetectors, and light emitting diodes. HAXPES and X-ray photoelectron spectroscopy (XPS) are effective techniques for elucidating the composition-electronic structure–property dependence at the bulk and surface of the thin films, respectively. Synchrotron-based HAXPES and XPS have been utilized to confirm the presence of Pb^0^ in perovskite films.^18−21^ XPS is highly surface-sensitive; however, HAXPES (hard X-rays, i.e., photon energies more than 2 keV) is a minimally destructive technique used to probe the bulk-like properties of the perovskite films since higher photon energies increase the inelastic mean free path of the photoelectrons emitted from a specific core level and, hence, results in information being obtained from a greater depth. In the manuscript, the word “bulk” has been used to mean “bulk-like” or “sub-surface” regions within the top ∼50 nm of the surface.

In this study, a laboratory-based HAXPES spectrometer with Ga Kα (9.25 keV) X-ray source was used to investigate the compositional heterogeneity in the bulk and at the surface of CsPbIBr_2_ perovskite films fabricated without and with antisolvent engineering. This novel HAXPES system was recently used to probe the contribution of substituted alkali-metal cations in the improved device performance and stability of hybrid halide perovskite devices but has not been explored in the case of inorganic halide perovskite semiconductors.^23^ In this study, the presence of Pb^0^, and its varying distribution at the surface and in the bulk was investigated by combining the strengths of lab-based HAXPES and soft X-ray photoemission methods to unravel the difference in distribution of metallic Pb^0^ defects depending on the use of different antisolvents and shed new insight into the ambient stability of CsPbIBr_2_ perovskite-based photovoltaic devices. Our investigation shows that the concentration of metallic Pb^0^ can be higher in the bulk than at the surface and can be eliminated by the use of appropriate antisolvents to yield ambient stable CsPbIBr_2_ films with better photovoltaic properties.

## 2 Experimental Section

### 2.1 Materials

The CsI and PbBr_2_ (99.999% purity) perovskite precursors were purchased from Alfa Aesar. The solvent dimethyl sulfoxide (DMSO, anhydrous, ≥99.9%) and antisolvents, chlorobenzene, and diethyl ether (anhydrous, ≥99.7%), used to prepare the CsPbIBr_2_ perovskite solution and films were purchased from Sigma-Aldrich.

### 2.2 Film Fabrication

Nonpatterned indium tin oxide (ITO) coated glass substrates with a sheet resistance of 15 Ω □^–1^ were used as the substrates for solar cell fabrication. ITO substrates were cleaned sequentially by ultrasonication in sodium dodecyl sulfate (SDS) solution, deionized water, acetone, and isopropyl alcohol. The substrate cleaning process was completed by plasma cleaning with oxygen plasma for 3 min in a Plasma Asher (MiniFlecto, Gala Instrumente GmbH). For the CsPbIBr_2_ perovskite precursor solution, 1 M CsI (259.8 mg) and 1 M PbBr_2_ (367.01 mg) were mixed in 1 mL of anhydrous DMSO and stirred continuously for 2 h at room temperature. For the deposition of the CsPbIBr_2_ films, a two-step spin-coating procedure was followed: the first step at 1000 rpm for 15 s at an acceleration of 1000 rpm sec^–1^ followed by a second step at 4000 rpm for 45 s at an acceleration of 2000 rpm sec^–1^. Finally, 750 μL of antisolvents (chlorobenzene and diethyl ether) was poured during the last 15^th^ second of the second spin coating step. After spin coating, the films were annealed at 150 °C on a hot plate for 15 min; the perovskite films turned reddish orange immediately upon placing them on the hot plate, indicating the formation of photoactive perovskite α-phase CsPbIBr_2_. The entire sequence of film fabrication including perovskite precursor weighing, precursor solution preparation, stirring, and perovskite active layer spin coating was undertaken inside a nitrogen-filled glovebox with H_2_O and O_2_ level <0.1 ppm.

### 2.3 Characterization of CsPbIBr_2_ Perovskite Films

Hard X-ray Photoelectron Spectroscopy (HAXPES) measurements were performed using monochromated Ga Kα X-ray radiation (9252 eV, 3.57 mA emission at 250 W, microfocused to 50 μm) and an EW-4000 high voltage electron energy analyzer (HAXPES-Lab, Scienta Omicron GmbH); the instrument has a base vacuum pressure of ∼5 × 10^–10^ mbar.^24,25^ The entrance slit width used was 0.8 mm, and the pass energies used for survey and core level spectra were 500 and 200 eV respectively, with energy resolutions of 1.2 and 0.6 eV.^24^ The HAXPES instrument also has a monochromated Al Kα X-ray source (1486 eV, 20 mA emission at 300 W) for surface-sensitive XPS at the same sample position (with an energy resolution of ∼0.3 eV). A low-energy electron flood source (FS40A, PreVac) was used for realizing charge neutralization for insulating samples. Binding energy scale calibration was performed with respect to the Cs 3*d*_5/2_ core levels at 725 eV. Analysis and curve fitting were performed using Shirley backgrounds with GL(50) Voigt-approximation peaks using CasaXPS.^26^ Core level relative sensitivity factors for HAXPES quantification were calculated according to these refs (25,27).

The surface morphology of the prepared CsPbIBr_2_ perovskite films was studied by using a scanning electron microscope (Hitachi S4800 equipment). The thickness of the CsPbIBr_2_ perovskite films (∼240 nm) was measured using a Veeco Dektak 150 profilometer. The XRD patterns of the films were acquired in the range of 10°–60° with a step size of 0.017° using a Panalytical Empyrean X-ray diffractometer with Cu Kα_1_ radiation.

## 3 Results and Discussion

### 3.1 Microstructural Characterization of the CsPbIBr_2_ perovskite films

**Figure 1:**
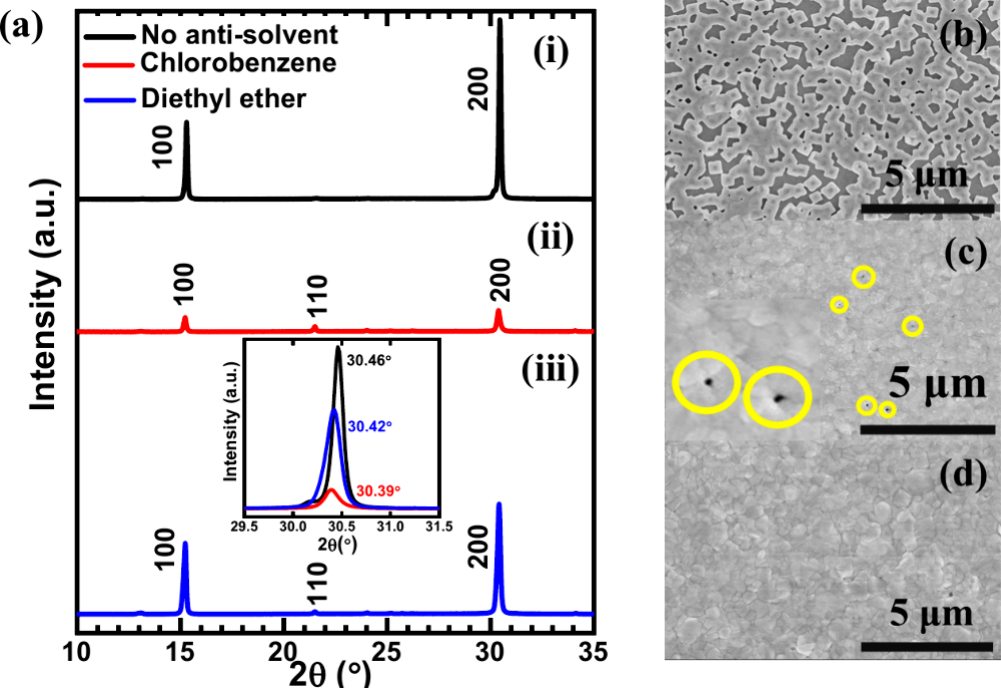
(a) (i–iii) X-ray diffractograms of the CsPbIBr_2_ perovskite films fabricated (i) without antisolvent, with (ii) chlorobenzene, and (iii) diethyl ether treatment (all graphs have been drawn to the same scale bar). The inset in Figure 1(a) iii shows the peak shift for the prominent orientation of the (200) crystal plane for the corresponding samples. (b–d) Secondary electron images of CsPbIBr_2_ films with (b) no antisolvent treatment, (c) chlorobenzene (presence of pinholes shown as yellow circles and inset shows the pinholes at higher magnification), and (d) diethyl ether treatment, obtained using scanning electron microscopy.

X-ray diffraction measurements were undertaken to investigate the crystallinity of the CsPbIBr_2_ perovskite films without antisolvent and with chlorobenzene and diethyl ether treatments. Figure 1(a) shows the characteristic peaks at 15.24°, 21.52°, and 30.42° corresponding to the (100), (110), and (200) planes of the cubic α-phase CsPbIBr_2_. The obtained XRD pattern matches the previous reports for CsPbIBr_2_ perovskite films.^15,28−30^ However, the intensity of the X-ray diffraction was lowest in the case of CsPbIBr_2_ films treated with chlorobenzene antisolvent [Figure 1 (a) (ii)] which implies poor crystalline properties compared to the pristine CsPbIBr_2_ films (prepared without any antisolvent) and with diethyl ether treatment [Figure 1 (a) (i) and Figure 1 (a) (iii), respectively]. Further, a closer view of the 2θ value [(Figure 1a(iii) inset)] corresponding to the prominent crystal plane orientation of (200) has revealed a shift to lower diffraction angle for the antisolvent treated CsPbIBr_2_ films compared to the one without any treatment (from 30.46° to 30.39°). The highest shift has been observed for the CsPbIBr_2_ films with diethyl ether treatment, suggesting the crystal lattice expansion and possibly due to better mixing of iodine into the lattice. A further close look into the XRD spectra of CsPbIBr_2_ perovskite films without antisolvent and with diethyl ether treatment shows an additional peak (of intensity nearly equal to the (110) peak) at 2θ of around 13° and this can be attributed to PbI_2_ as per the previous publications.^31−33^

The secondary electron (SE) surface morphology of the CsPbIBr_2_ films was acquired using scanning electron microscopy as shown in parts b–d. CsPbIBr2 films fabricated without any antisolvent treatment showed the formation of a discontinuous cubic-shaped domain appearance which lacks surface coverage [Figure 1(b)]. Dense and compact films were formed after antisolvent treatment; however, the presence of pinholes in the chlorobenzene treated films was visible [Figure 1(c), pinholes highlighted in yellow]. Treatment of CsPbIBr_2_ films with diethyl ether resulted in the best surface morphology having compact and uniform grain-like features as shown in Figure 1(d).

### 3.2 Stability of the Glass/ITO/SnO_2_/CsPbIBr_2_/Spiro-OMeTAD/Au Device Stack

**Figure 2:**
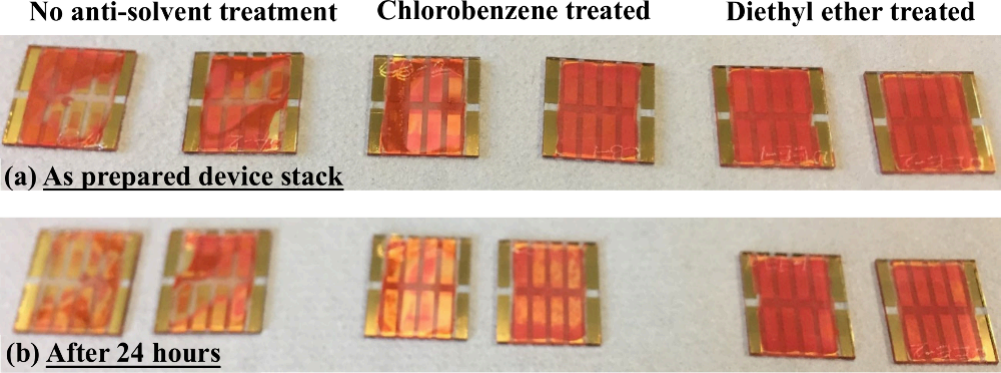
Photographs of the Glass/ITO/SnO_2_/CsPbIBr_2_/Spiro-OMeTAD/Au photovoltaic device stack without antisolvent and with chlorobenzene and diethyl ether treatment in (a) as-prepared condition and (b) after aging at 30% relative humidity in a desiccator for 24 h.

In the present study, the as-prepared Glass/ITO/SnO_2_/CsPbIBr_2_/Spiro-OMeTAD/Au device stack was stored in a desiccator at a relative humidity of 30% overnight and the photographs of the freshly prepared devices and after 24 h of aging were recorded [Figure 2(a) and (b), respectively]. As can be observed from the figure, the loss in the reddish-orange color of the photovoltaic devices comprising the CsPbIBr_2_ films without any antisolvent washing and with chlorobenzene treatment signifies the degradation of the perovskite layer, whereas the diethyl ether treated films showed no degradation even after 24 h. According to a previous study conducted by the authors, antisolvent engineering using diethyl ether reportedly resulted in a reduction in defect density and grain misorientation in the CsPbIBr_2_ perovskite films and an improvement in the stability of corresponding photovoltaic devices.^14^ Photovoltaic performance parameters of the devices comprising CsPbIBr_2_ perovskite films with diethyl ether treatment recorded superior performance and stability (champion power conversion efficiency of 5.9% under 1 Sun) as compared to the devices fabricated with CsPbIBr_2_ films without any antisolvent engineering and with chlorobenzene treatment (power conversion efficiencies of 5.9% and 3.0%, respectively), with the latter techniques resulting in accelerated device degradation during 1 Sun photovoltaic performance measurement [Figure S1 of the Supporting Information].^14^ The presence of pinholes and the discontinuous morphology in the CsPbIBr_2_ films without antisolvent treatment and with chlorobenzene treatment can contribute to their poor device performance and lack of stability. Since the presence of Pb^0^ can cause shunting paths for accelerated degradation,^21^ the variation in the Pb^0^ content and composition at the bulk and the surface of the CsPbIBr_2_ perovskite films were probed using XPS and HAXPES, to unravel the role of Pb^0^ and phase instability of CsPbIBr_2_ films as discussed in the following sections.

### 3.3 Compositional Analysis Using Hard X-ray Photoelectron Spectroscopy (HAXPES)

**Figure 3:**
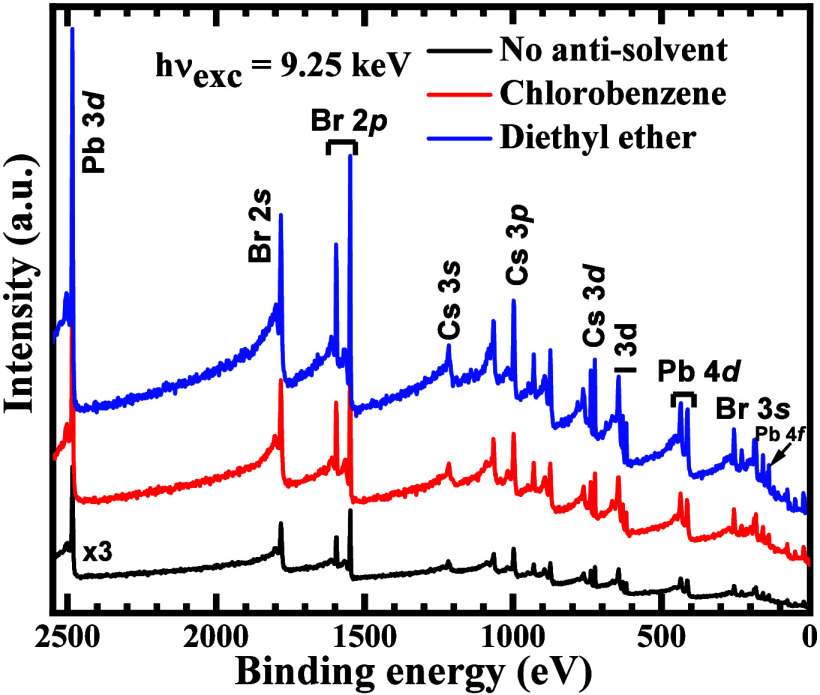
Wide/survey spectra of CsPbIBr_2_ perovskite films prepared without and with chlorobenzene and diethyl ether antisolvent treatment probed using HAXPES.

Figure 3 shows the survey spectra of the pristine and antisolvent treated CsPbIBr_2_ perovskite films probed using HAXPES. The presence of the core levels of the metallic components including Pb 4*f*, Pb 4*d*, Pb 3*d*, Cs 3*d*, Cs 3*p*, and Cs 3*s* as well as the halide components (Br 3*s*, Br 3*p*, and I 3*d*) were observed in the survey spectra. The spectra were aligned with respect to the Cs 3*d*_5/2_ core level at 725 eV.^23^ The sampling depth for HAXPES and XPS was calculated according to the literature.^34,35^ For HAXPES (9252 eV photon energy), the sampling depth is calculated as three times the inelastic mean free path of the photoelectrons, and that for the Pb 4*f* and Pb 3*d* core levels were determined to be 47 and 36 nm, respectively. The corresponding sampling depth for soft XPS using a 1486 eV Al Kα source was determined as 10 nm.

**Figure 4:**
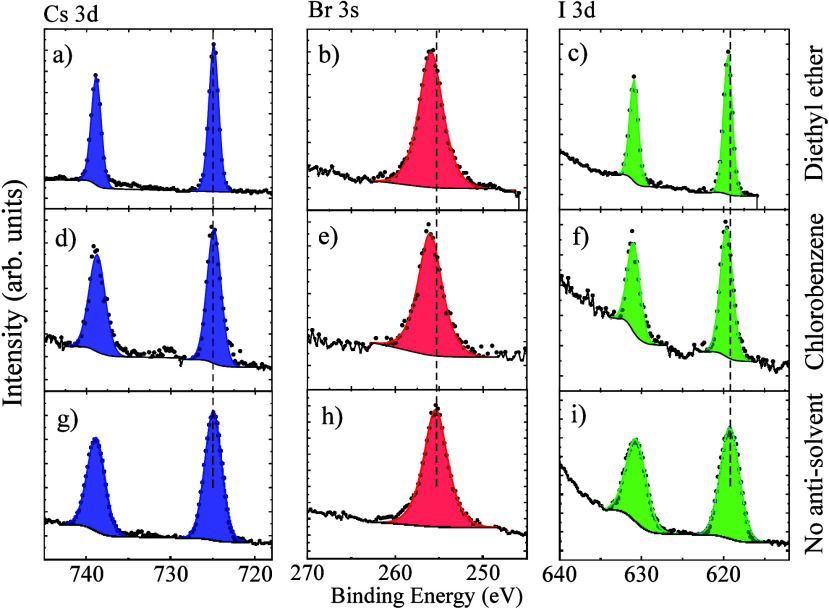
High resolution HAXPES core level spectra of Cs 3*d* [(a), (d), & (g)], Br 3*s* [(b), (e), & (h)] and I 3*d* [(c), (f), & (i)] from pristine and antisolvent treated CsPbIBr_2_ films.

Figure 4 shows the comparison of the high-resolution Cs 3*d*, Br 3*s*, and I 3*d* core level spectra of the CsPbIBr_2_ films fabricated without antisolvent engineering and with chlorobenzene and diethyl ether treatment. The doublet feature at 725.5 ± 0.2 eV and 739.1 ± 0.2 eV can be attributed to Cs 3*d*_5/2_ and Cs 3*d*_3/2_ with a spin–orbit splitting of 13.9 eV [Figure 4 (a), (d), and (g)] whereas the peaks at 619.8 ± 0.2 eV and 631.3 ± 0.2 eV can be assigned to I 3*d*_5/2_ and I 3*d*_3/2_ (splitting of 11.5 eV) [Figure 4 (c), (f), and (i)].^23^ The peak at ∼256 eV can be attributed to Br 3*s*^36^ [Figure 4(b), (e), and (h)]. Treatment with different antisolvents results in two observable changes for the I 3*d*_5/2_ peak (a) shift to higher binding energy values and (b) narrowing of the full-width half-maximum (fwhm) of the Cs 3*d* and I 3*d* peaks. The narrowing of the fwhm can be related to the improved microstructure of the antisolvent treated CsPbIBr_2_ films (such as uniform film formation and surface coverage) compared to the untreated films as shown in [Figure 1(b)–(d)].^37^ The Br 3*s* peak also shows a shift to higher binding energy values upon the antisolvent treatment, similar to the I 3*d*_5/2_ peak, implying the better chemical bonding of the iodine and bromine to the lead and better halide incorporation to the bulk-like regions of CsPbIBr_2_ films.^38^ The better incorporation of iodine to the CsPbIBr_2_ films upon antisolvent washing is in close agreement with the 2θ shift in the XRD pattern as shown in Figure 1(a).

**Figure 5:**
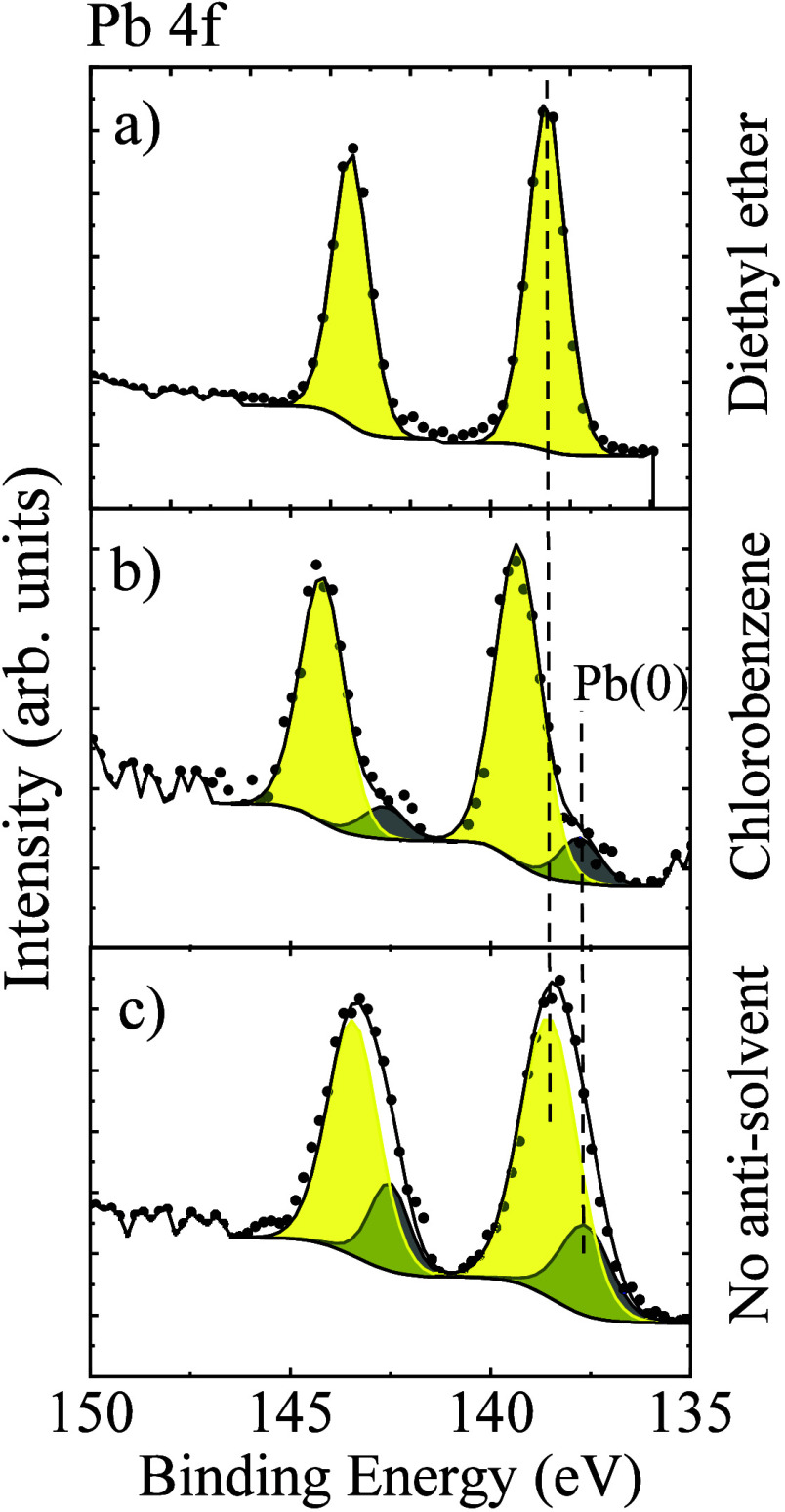
High resolution HAXPES core level spectra of Pb 4*f* from CsPbIBr_2_ films prepared (a) without antisolvent treatment, (b) with chlorobenzene, and (c) diethyl ether treatment, respectively.

Figure 5 shows the Pb 4*f* spectra for the films and consist of Pb 4*f*_7/2_ and Pb 4*f*_5/2_ doublet feature at 138.4 ± 0.1 eV and 143.1 ± 0.1 eV, respectively, with the primary Pb 4*f*_7/2_ peak at 138.4 ± 0.1 eV, corresponding to the Pb^2+^ in the perovskite structure.^21,39^ The peaks corresponding to Pb^0^ were observed at ∼136.9 ± 0.2 and 141.8 ± 0.2 eV assigned to Pb^0^ 4*f*_7/2_ and Pb^0^ 4*f*_5/2_, respectively, in all samples except the diethyl ether treated samples [Figure 5(a)]. The Pb^0^ component has often been considered similar to defect or trap states and recombination centers for charge carriers which detrimentally affect the corresponding perovskite device performance and stability.^21^ The absence of Pb^0^ related peak in the Pb 4*f* spectra from the bulk-like region of the diethyl ether treated CsPbIBr_2_ films thus accounts for the better photovoltaic properties and improved stability of the corresponding devices. This is in agreement with the recent study by Liang et al.^40^ in which the authors also reported much better photovoltaic efficiency and stability for the perovskite devices without detectable Pb^0^ impurity [SI Figure S1]. For the chlorobenzene antisolvent treated CsPbIBr_2_ films, the Pb 4*f*_7/2_ peak shifts to higher binding energy values indicating the introduction of more I^–^ ions and changes in the chemical bonding,^41^ which is in agreement with the highest shift to larger 2θ values in the XRD pattern as shown in Figure 1(a).

As can be observed from Figure 5, the presence of Pb^0^ is found in the CsPbIBr_2_ films, without antisolvent treatment and chlorobenzene-treated samples, however, the fraction of the Pb^0^ component in the Pb 4*f* core level shows a significant variation. The diethyl ether treated CsPbIBr_2_ films did not show any detectable traces of Pb^0^. The quantification was performed after spectral decomposition of the Pb 4*f* doublet using CasaXPS with the residue χ^2^ < 0.5 indicating an adequate peak fit. The fraction of Pb^0^ was calculated from its relative intensity in the spectra, and this was reduced to negligibly small (not detectable) after treatment with diethyl ether [atomic% of Pb^0^ ∼ 0%] compared to when no solvent engineering was performed [atomic% ∼ 21%]. The absence of Pb^0^ in the bulk-like region after diethyl ether treatment suggests fewer defects and improved quality of the corresponding films and corroborates the best photovoltaic device performance in diethyl ether treated CsPbIBr_2_ films as reported previously by the authors^14^ (shown in Figure S1) and by Liang et al.^40^ The fast degradation of the devices with no-antisolvent and chlorobenzene treated CsPbIBr_2_ films can be attributed to the high defect density in the form of Pb^0^ component [atomic% ∼ 21% & 10.7%, respectively] in such films. The evolution of Pb^0^ is often associated with the loss of iodine from the perovskite lattice^42^ and hence, the absence of Pb^0^ component in the diethyl ether-treated CsPbIBr_2_ films suggests better stoichiometric formation of the perovskite films.

It is worth noting that previous studies have shown that metallic Pb° can be formed due to the radiolysis of PbX_2_ present in the halide perovskite film under the high-intensity synchrotron-based HAXPES.^40,43^ The PbX_2_ in these samples were formed either due to the deliberate aging or the high bright light illumination. In the present study, such effects are considered negligible as the samples are freshly prepared (not deliberately aged), not exposed to high bright lights and the intensity of the HAXPES source is 3–4 orders of magnitude lower than the synchrotron-based source. Also, as explained in the XRD pattern of these CsPbIBr_2_ films, the samples without antisolvent and with diethyl ether treatment have a very tiny peak around 2θ ∼13° which can be attributed to the PbI_2_, but no such peak has been seen for CsPbIBr_2_ films treated with chlorobenzene. However, the amount of metallic Pb^0^ detected in these CsPbIBr_2_ films is not aligned with this observation: diethyl ether-treated CsPbIBr_2_ sample has the minimum metallic Pb^0^ and the nonantisolvent treated CsPbIBr_2_ sample has the highest content. This also indicates that the observed metallic Pb^0^ in the present study is not correlated with the presence of PbX_2_ initially present in the films.

### 3.4 Compositional Analysis Using the XPS

**Figure 6:**
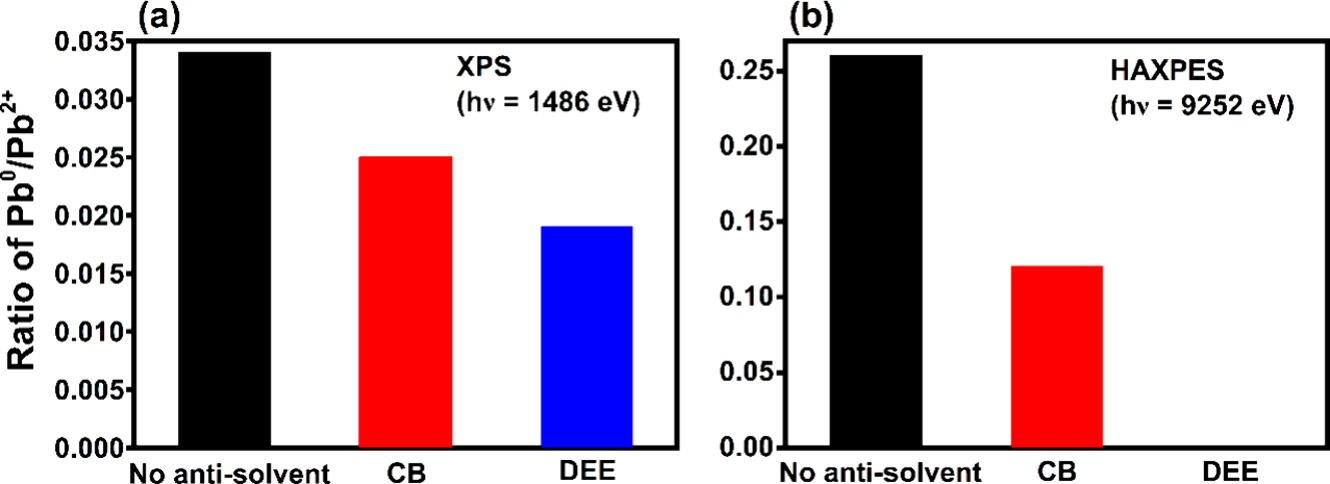
Variation in the ratio of Pb^0^/Pb^2+^ due to antisolvent treatment probed using (a) XPS and (b) HAXPES.

In an earlier study, the authors have shown the variation in Pb^0^ at the surface of the CsPbIBr_2_ perovskite films as a result of antisolvent treatment with chlorobenzene and diethyl ether, probed using XPS.^14^ The corresponding plot is shown in Figure S2. The area under the fitted curve for the Pb 4*f* core level corresponding to the Pb^0^ component is observed to have decreased because of antisolvent treatment suggesting a reduction in defect concentration (Table S1). Figure 6 shows the variation in the ratio of Pb^0^/Pb^2+^ due to antisolvent treatment probed using (a) XPS and (b) HAXPES. On the surface of the CsPbIBr_2_ films, the Pb^0^/Pb^2+^ fraction reduced from ∼0.034 for films without antisolvent treatment to ∼0.019 upon treatment with diethyl ether [Figure 6(a) and Table S1]. In the current study, HAXPES shows a significant variation in the Pb^0^ content upon treatment with antisolvent [∼0.26, ∼0.12, and ∼0 for films without antisolvent, with chlorobenzene and diethyl ether treatment, respectively] [Figure 6(b)]. These results suggest substantial heterogeneity in the Pb^0^ content in the vertical direction of the films with values being very low at the surface (within 10 nm) compared to that in the bulk-like region (∼47 nm) for pristine CsPbIBr_2_ samples and with chlorobenzene treatment. The higher concentration of Pb^0^ in the bulk of perovskite films has been previously reported in the literature and suggests a higher density of defects further into the bulk-like material than at the surface.^21^

The CsPbIBr_2_ films without antisolvent treatment and with chlorobenzene treatment show the highest content of Pb^0^ in the bulk region and the highest vertical heterogeneity in the surface vs bulk Pb^0^ (∼five times in the bulk compared to the surface), whereas after diethyl ether treatment Pb^0^ impurity could not be detected at all in the bulk-like region of the corresponding films. As shown in Figure 2 and discussed in section 3, devices fabricated using chlorobenzene treated and without antisolvent-treated CsPbIBr_2_ films show the fastest degradation or largest phase instability, although initially the corresponding devices show similar photovoltaic performance under 1 Sun illumination. The higher binding energy shift of the Pb 4*f*_7/2_ peak, the largest XRD peak shift to a higher 2θ value and the high content of Pb^0^ impurity for the chlorobenzene treated CsPbIBr_2_ films imply the possibility of excess PbI_2_, which can enhance the perovskite photodegradation.^40^ Both the best photovoltaic device performance and the highest phase stability have been shown by the devices comprising the diethyl ether treated CsPbIBr_2_ films for which the least amount and least variation in the bulk-to-surface ratio of Pb^0^ are observed. Thus, a direct correlation between the phase stability of CsPbIBr_2_ devices and the vertical heterogeneity in Pb^0^ of the corresponding CsPbIBr_2_ films is revealed by combining XPS and HAXPES measurements.

### 3.5 Valence Band Spectra and Other Core Levels Measured Using HAXPES

**Figure 7:**
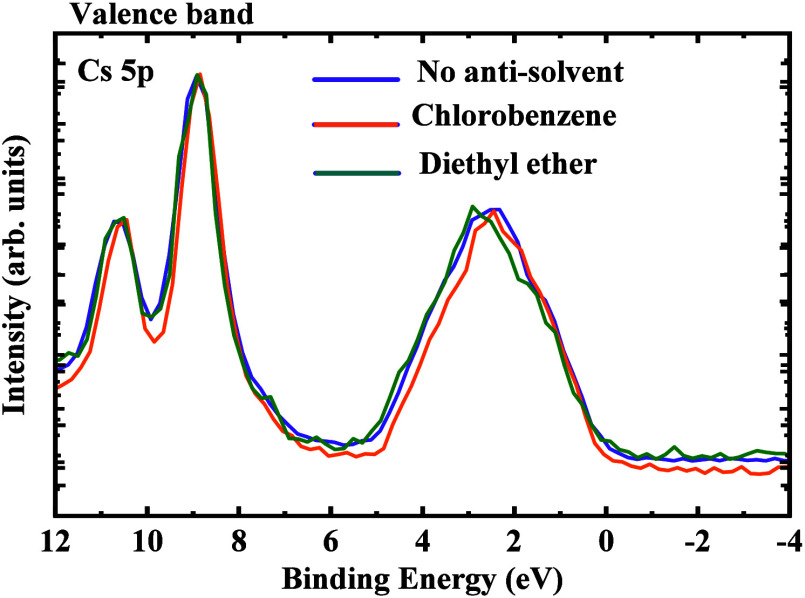
High-resolution HAXPES valence band spectra corresponding to the CsPbIBr_2_ films prepared without antisolvent, (ii) with chlorobenzene, and (iii) diethyl ether treatment.

Understanding the position of the valence band is of crucial importance in perovskite photovoltaic research for band alignment at the interfaces of the devices to improve charge extraction and device performance. Using hard X-rays to probe the valence band allows for an understanding of the electronic structure at tens of nanometers of analysis depth and hence is useful in investigating more bulk-like properties in the perovskite films. Figure 7 shows the high-resolution valence band spectra of the CsPbIBr_2_ films prepared without antisolvent, with chlorobenzene, and diethyl ether treatment. The spectral region around 2–6 eV can be attributed predominantly to Pb 6*s* and I 5*p* hybridized states.^39^ The lack of an O 2*p* signature around 8 eV signifies the absence of oxygen in the all-inorganic CsPbIBr_2_ perovskite. The doublet feature between 9.5 and 13 eV can be assigned to the relatively shallow core level corresponding to Cs 5*p*.^44^ There is no observable variation in the position of the valence band maxima due to the antisolvent treatment of the perovskite layer implying that the antisolvent treatment does not result in any compositional or stoichiometric variation in the films.

## 4 Conclusions

In summary, for the first time, the chemical composition in the bulk-like region of CsPbIBr_2_ perovskite films was investigated using HAXPES and contrasted with that of the corresponding surface chemical composition revealing a striking vertical heterogeneity in Pb^0^ impurity content. Further, this study revealed a direct correlation between the amount and vertical distribution heterogeneity of the Pb^0^ impurity and the photovoltaic performance and stability losses in all-inorganic CsPbIBr_2_ perovskites. The photovoltaic devices made with CsPbIBr_2_ films having below detectable levels of Pb^0^ impurity demonstrated the best power conversion efficiency and stability under ambient conditions. This study concluded that the amount of Pb^0^ impurity in CsPbIBr_2_ perovskites can be reduced by engineering the film growth using appropriate antisolvents. This work highlights the application of hard and soft X-ray spectroscopy in understanding the role of microstructural and elemental defect/trap density in CsPbIBr_2_ perovskite films and establishes a protocol for mitigating the presence of Pb^0^ defects and enhancing the photovoltaic properties and phase stability by surface engineering using appropriate antisolvent treatment.

## Data Availability

The research data underpinning this publication can be accessed at 10.17630/40f00045-224b-4a51-bba7-770194baeb97 [REF].

## Supporting Information Available

Photovoltaic performance comparison for CsPbIBr_2_ devices with and without antisolvent treatment, high-resolution XPS core level spectra of Pb 4*f* from CsPbIBr_2_ films including the metallic lead (Pb^0^ 4f), and a table listing the atomic % and Pb^0^/Pb^2+^ ratio in case of the CsPbIBr_2_ films (PDF)
