# Supplementary material for: Vertical Compositional Heterogeneity Induces Instability in All-Inorganic CsPbIBr2 Perovskites
Source: ACS Appl Energy Mater. 2024 Oct 8;7(20):9045–51. doi: 10.1021/acsaem.4c01898 (PMC11523065; doi:10.1021/acsaem.4c01898)
Supplement: Supplementary file 1 — ae4c01898_si_001.pdf [file ae4c01898_si_001.pdf]

Supporting Information

Vertical compositional heterogeneity induces instability in all-inorganic $CsPbIBr_2$ perovskites

Paheli Ghosh[a], Ben F. Spencer[b], and Lethy Krishnan Jagadamma[a*]

[a]Energy Harvesting Research Group, School of Physics & Astronomy, SUPA, University of St Andrews, St Andrews, KY16 9SS, United Kingdom

[b]Henry Royce Institute and Department of Materials, the University of Manchester, Oxford Road, Manchester, M13 9PL, United Kingdom

Email: lkj2@st-andrews.ac.uk

Supporting Information

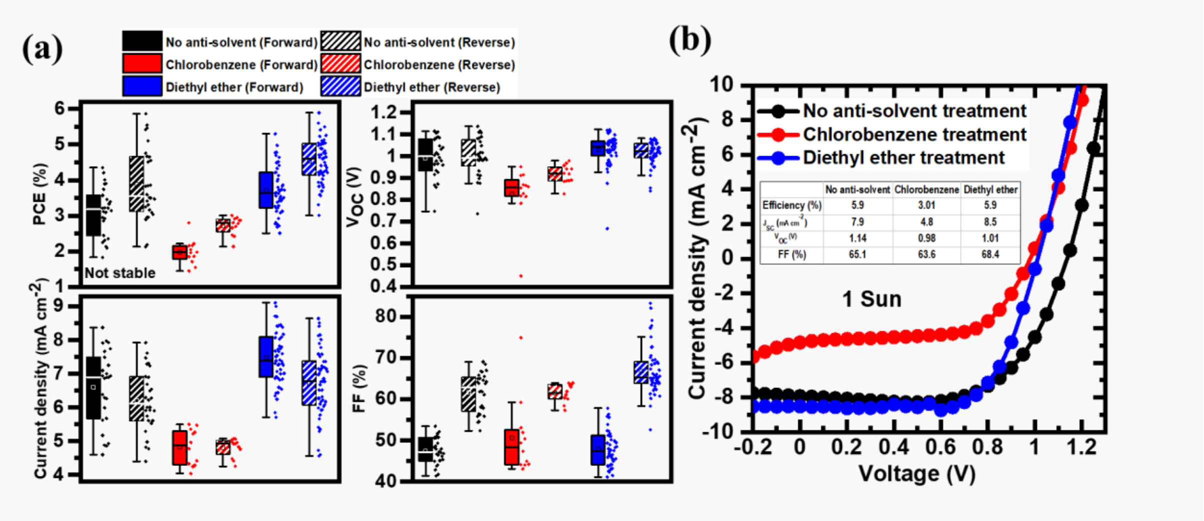

Figure S1: Photovoltaic performance comparison. (a) (Clockwise from top left) Distribution of photovoltaic parameters PCE (%), V_{OC} (V), FF (%) and J_{SC} (mA cm^{-2}) for $CsPbIBr_2$ devices fabricated without anti-solvent treatment, with chlorobenzene, and diethyl ether in forward and reverse scans under 1 Sun. (b) Current density *vs* voltage curves for the best-performing devices with no anti-solvent treatment, chlorobenzene, and diethyl ether treatment along with the respective photovoltaic parameters under 1 Sun in reverse scan. [reproduced with permission from the authors' previous work[1]].

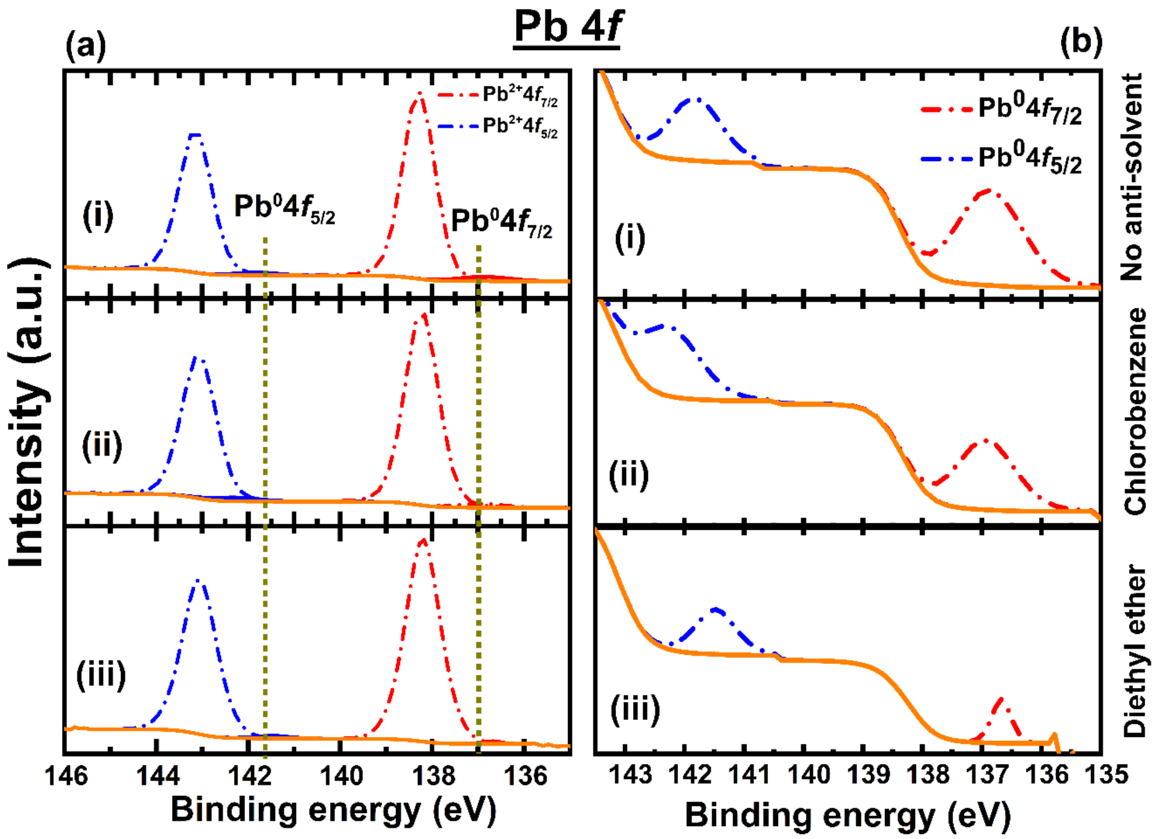

Figure S2: (a) High-resolution XPS core level spectra of Pb 4*f* from $CsPbIBr_2$ films prepared (i) without anti-solvent treatment, (ii) with chlorobenzene, and (iii) diethyl ether treatment, respectively, and (b) the XPS spectra of Pb 4*f* showing the metallic lead (Pb^0 4*f*) components in the films prepared (i) without anti-solvent treatment, (ii) with chlorobenzene, and (iii) diethyl ether treatment, respectively.

Table S1: Atomic % and Pb^0/Pb^{2+} ratio in case of the $CsPbIBr_2$ films prepared (i) without anti-solvent treatment, (ii) with chlorobenzene, and (iii) diethyl ether treatment calculated from CasaXPS fitting.

	XPS			HAXPES		
	No anti-solvent	Chloro benzene	Diethyl ether	No anti-solvent	Chloro benzene	Diethyl ether
Atomic % of Pb^0 ± 0.2	3.27	2.43	1.9	21.0	10.7	0
Pb^0/Pb^{2+} ratio (%)	0.034	0.025	0.019	0.26	0.12	0

1. Ghosh, P., Bruckbauer, J., Trager-Cowan, C. & Krishnan Jagadamma, L. Crystalline grain engineered CsPbIBr2 films for indoor photovoltaics. *Appl. Surf. Sci.* **592**, 152865 (2022).
